# Supplementary material for: Empirical methods for controlling false positives and estimating confidence in ChIP-Seq peaks
Source: BMC Bioinformatics. 2008 Dec 5;9:523. doi: 10.1186/1471-2105-9-523 (PMC2628906; doi:10.1186/1471-2105-9-523)
Supplement: Additional File 1 — A variety of html documents from the USeq web site detailing the available applications, their best usage, output file type descriptions, command line menus, etc. [file 1471-2105-9-523-S1.zip › USeqUserGuides/JavaDoc.zip!/JavaDoc/trans/misc/InefficientPseudoMedian.html]

InefficientPseudoMedian


|  |  |  |
| --- | --- | --- |
| **Overview** \| **Package** \| **Class** \| **Use** \| **Tree** \| **Deprecated** \| **Index** \| **Help** | |  |
| **PREV CLASS**   **NEXT CLASS** | **FRAMES**    **NO FRAMES**     **All Classes** |  |
| SUMMARY: NESTED \| FIELD \| CONSTR \| METHOD | DETAIL: FIELD \| CONSTR \| METHOD |  |


---


## trans.misc Class InefficientPseudoMedian

```
java.lang.Object
  trans.misc.InefficientPseudoMedian
```

---

``` public class InefficientPseudoMedian extends java.lang.Object ```

---

**Constructor Summary**

`InefficientPseudoMedian()`


| **Method Summary** | |
| --- | --- |
| `void` | `cleanUpHash(int newStartTwo)`             Removes Pairs from the hash or reinstantiates if not needed. |
| `static void` | `main(java.lang.String[] args)` |
| `static void` | `standard(java.lang.String[] args)` |
| `static int[]` | `totalNumberRatiosByWindowSize(int numOligoRatios, int maxWindowSize)`             Precaluculates the total number of pairwise ratios for a range of window sizes. |

**Methods inherited from class java.lang.Object**

`equals, getClass, hashCode, notify, notifyAll, toString, wait, wait, wait`

**Constructor Detail**

### InefficientPseudoMedian

```
public InefficientPseudoMedian()
```


**Method Detail**

### cleanUpHash

```
public void cleanUpHash(int newStartTwo)
```

:   Removes Pairs from the hash or reinstantiates if not needed.

---


### totalNumberRatiosByWindowSize

```
public static int[] totalNumberRatiosByWindowSize(int numOligoRatios,
                                                  int maxWindowSize)
```

:   Precaluculates the total number of pairwise ratios for a range of window sizes.
    The index is the window size 0,1,2,3... maxWindowSize.

---


### main

```
public static void main(java.lang.String[] args)
```

---


### standard

```
public static void standard(java.lang.String[] args)
```


---


|  |  |  |
| --- | --- | --- |
| **Overview** \| **Package** \| **Class** \| **Use** \| **Tree** \| **Deprecated** \| **Index** \| **Help** | |  |
| **PREV CLASS**   **NEXT CLASS** | **FRAMES**    **NO FRAMES**     **All Classes** |  |
| SUMMARY: NESTED \| FIELD \| CONSTR \| METHOD | DETAIL: FIELD \| CONSTR \| METHOD |  |


---
